# Supplementary material for: Assessment of phytochemical screening, antibacterial, analgesic, and antipyretic potentials of Litsea glutinosa (L.) leaves extracts in a mice model
Source: PLoS One. 2025 Jan 31;20(1):e0309857. doi: 10.1371/journal.pone.0309857 (PMC11785304; doi:10.1371/journal.pone.0309857)
Supplement: Attachment — Submitted filename: Review Report for Manuscript Number PONE-D-24-35607.docx [file pone.0309857.s001.docx]

**Review Report for Manuscript Number PONE-D-24-35607**

Manuscript is written scientifically well but need some minor revisions of grammatical mistakes as well formatting mistakes as some of them are mentioned below.

Unit for gram should be used consistently

Re-write line 187-188

Line-190; tape water should be replaced with tap water

In section 2.14 scientific names of bacteria should be italic

Figure number should be reviewed as fig 1a and 3A showed different format

In table 6, salmonella should be started with capital letter

The quality of all the pictures is not satisfactory, it should be improved

After correction of minor revisions this manuscript can be accepted for publication in PLOS ONE.
