# Supplementary material for: Assessment of phytochemical screening, antibacterial, analgesic, and antipyretic potentials of Litsea glutinosa (L.) leaves extracts in a mice model
Source: PLoS One. 2025 Jan 31;20(1):e0309857. doi: 10.1371/journal.pone.0309857 (PMC11785304; doi:10.1371/journal.pone.0309857)
Supplement: Attachment — Submitted filename: Response to Reviewers.docx [file pone.0309857.s002.docx]

We sincerely appreciate the reviewers' and the editor’s thorough evaluation of our manuscript titled *“Assessment of Phytochemical Screening, Antibacterial, Analgesic, and Antipyretic Potentials of Litsea glutinosa (L.) Leaves Extracts in a Mice Model”* (PONE-D-24-35607). We have carefully considered all comments and have revised the manuscript accordingly. Below, we provide a detailed response to each reviewer’s feedback.

**Reviewer #1:**

1. **Unit for gram should be used consistently**
   **Response**: We have reviewed the manuscript and ensured that the unit for "gram" is used consistently throughout the manuscript.
1. **Re-write line 187-188**
   **Response**: The sentence in lines 187-188 has been rephrased for clarity and precision.
2. **Line-190; tape water should be replaced with tap water**
   **Response**: The error has been corrected, and "tape water" is now replaced with "tap water."
3. **In section 2.14, scientific names of bacteria should be italicized**
   **Response**: All scientific names in Section 2.14 have been properly italicized, following standard formatting for scientific nomenclature.
4. **Figure numbers (1a and 3A) should be reviewed for consistency**
   **Response**: We reviewed and standardized the figure formatting throughout the manuscript, ensuring consistent presentation of figure numbers.
5. **Capitalization of 'salmonella' in Table 6**
   **Response**: The term “salmonella” has been corrected to "Salmonella" in Table 6, using the appropriate capitalization.
6. **Improvement of picture quality**
   **Response**: We have improved the quality of all images in the manuscript, providing higher-resolution versions to enhance clarity and meet journal standards.

**Reviewer #2:**

1. **Grammar and typographical errors in the literature review section**
   **Response**: We have carefully revised the literature review section, addressing all typographical and grammatical errors. Recent articles have also been included to strengthen the background of the study.
2. **Correction of subheading 'Plant sample collection and accurate documentation'**
   **Response**: The subheading has been changed to “Sampling and Proper Documentation,” as suggested by the reviewer.
3. **Clarification on whether leaves or whole plant was collected**
   **Response**: We have clarified in the Methods section that only the plant leaves were collected for this study.
4. **Review of the Chemicals, Reagents, and Instruments section for sentence structure errors**
   **Response**: We thoroughly reviewed this section and corrected any sentence structure issues. The "Drying and Grinding of Plant Materials" and "Extraction" sections have also been improved for better readability.
5. **Typographical errors in the Results section**
   **Response**: All typographical errors in the Results section have been corrected. Additionally, the manuscript has undergone further review by a native English speaker to ensure grammatical accuracy.
6. **Improvement of the Discussion section and inclusion of recent articles**
   **Response**: We have added several recent articles to the Discussion section, strengthening the analysis of the results and providing better context for the findings. The figures and chromatograms have been enhanced to meet quality expectations.
7. **Reference list review and replacement of outdated/retracted references**
   **Response**: We have thoroughly reviewed the reference list to ensure that it is complete and correct. Outdated or predatory references have been removed and replaced with relevant current references. No retracted articles remain in the manuscript, but should any retracted articles need to be cited, we have ensured that their retracted status will be properly indicated in the References list. These changes are summarized in the attached reference corrections document.

**Reference Corrections:**

In response to the reviewers’ comments, we have made significant updates to the reference list. Outdated or predatory references have been replaced with credible, up-to-date sources. Additionally, we have added new references to reflect the most recent findings in the field. A detailed summary of these changes is provided in the attached **Reference Corrections** document.

We believe that these revisions address all the concerns raised by the reviewers, and we have significantly improved the manuscript based on their valuable feedback. We are grateful for the opportunity to revise our submission and look forward to your further comments.
