# Supplementary material for: Assessment of phytochemical screening, antibacterial, analgesic, and antipyretic potentials of Litsea glutinosa (L.) leaves extracts in a mice model
Source: PLoS One. 2025 Jan 31;20(1):e0309857. doi: 10.1371/journal.pone.0309857 (PMC11785304; doi:10.1371/journal.pone.0309857)
Supplement: Attachment — Submitted filename: Reviewer 1.docx [file pone.0309857.s003.docx]

Dear Editor,

I am pleased to inform that I have reviewed the revised manuscript no. PONE-D-24-35607R1 entitled Assessment of Phytochemical Screening, Antibacterial, Analgesic, and Antipyretic Potentials of Litsea glutinosa (L.) Leaves Extracts in a Mice Model. The authors have fully revised the article according to suggested modifications. Therefore, I recommend this article to be accepted in your esteemed Journal PLOS ONE.

Thanks.

Dr. Riffat Batool

Assistant Professor

Kohsar University Murree
